# Treatment of aortic aneurysms registered in Swedvasc: Development reflected in a national vascular registry with an almost 100% coverage

**DOI:** 10.1007/s00772-018-0414-8

**Published:** 2018-08-13

**Authors:** D. Bergqvist, K. Mani, T. Troëng, A. Wanhainen

**Affiliations:** 1 Department of Surgical Sciences, Vascular Surgery, Academic Hospital, 75185 Uppsala, Sweden; 2 Section of Vascular Surgery, Department of Surgical Sciences, Uppsala University, Uppsala, Sweden

**Keywords:** Vascular surgery, Vascular surgical procedures, Sweden/epidemiology, Population surveillance, Treatment outcome, Gefäßchirurgie, Gefäßchirurgische Verfahren, Schweden/Epidemiologie, Populationskontrolle, Behandlungsergebnis

## Abstract

Swedvasc is a registry for vascular surgical procedures, both open and endovascular. It was started in 1987 and since 1994 the whole population of Sweden is covered, at present around 10 million inhabitants. In a recent external validation, it was found to be highly accurate with abdominal aortic aneurysm surgery correctly reported in >96%. In this paper various factors explaining the almost 100% coverage are discussed, one important being that the registry has been developed and maintained within the profession of vascular surgery and not dictated by authorities. Another factor of importance is the possibility to use data in various research projects and so far 15 PhD theses have used Swedvasc data. To exemplify the practical use of the registry, the treatment of abdominal aortic aneurysms is scrutinized and among the various complications abdominal compartment syndrome is analyzed. Several significant temporal changes have been observed over the almost 25 years of Swedvasc: increasing use of endovascular surgery, treatment of aneurysms detected by screening , decreasing treatment for rupture, improved outcome, increasing treatment of older patients and patients with comorbid conditions. In conclusion, a high quality national vascular registry can be valid with high compliance and can be used to study population-based development of treatment and outcome. It can also be used to perform international comparisons with other registries, thereby getting an indication of the quality of care.

## Zusammenfassung

Swedvasc ist ein Register für alle in Schweden durchgeführten gefäßchirurgischen Eingriffe, sowohl offen als auch endovaskulär. Das Register wurde 1987 initiiert und umfasst von 1994 die ganze Bevölkerung von Schweden welche sich zurzeit auf ca. 10 Mio. Einwohner beläuft. Durch eine vor kurzem durchgeführte externe Validierung ist das Register als sehr präzise eingestuft worden. Das Bauchaortenaneurysma wurde in >96 % der Fälle korrekt dargestellt. Um die Praktikabilität des Registers zu evaluieren, ist die Behandlung von abdominellen Aortenaaneurysmen im Detail untersucht worden und neben anderen Komplikationen der Operation wurde das abdominelle Kompartmentsyndrom analysiert. In der vorliegenden Arbeit werden unterschiedliche Faktoren diskutiert, die für die fast 100 %ige Genauigkeit verantwortlich sind. Ein wichtiger Faktor ist, dass das Register von der gefäßchirurgischen Gesellschaft entwickelt und gepflegt worden ist und nicht von Behörden vorgelegt wurde. Ein anderer bedeutender Faktor ist die Möglichkeit, die Datei für unterschiedliche Forschungszwecke zu nutzen. Aktuell sind z.B. 15 Habilitationen, welche auf dieser Datei basieren. Im Verlauf der fast 25 Jahre in denen Swedvasc existiert ist es zu einer Anzahl von Veränderungen in der Diagnostik, den Behandlungsstrategien und in den Resultaten gekommen: mehr Verwendung von endovaskulären Techniken, Behandlung von Aneurysmen entdeckt durch Screening, fallende Tendenz für die Behandlung von Rupturen, sinkende postoperative Mortalität, mehr Behandlung von älteren Patienten und Patienten mit mehreren Komorbiditäten. Zusammenfassend kann ein nationales Gefäßregister sehr valide sein mit einer hohen Compliance. Das Register kann auch verwendet werden, um die eigenen nationalen Ergebnisse mit internationalen Ergebnissen von anderen Registern zu vergleichen. Letzteres würde dann u.a. eine Aussagekraft über die Qualität ermöglichen.

## Background

The population-based Vascular Registry in Southern Sweden (VRISS) was started in 1987, covering a population of 1.7 million inhabitants and with a 1-year follow-up, the first vascular registry with that aim [32]. Over time more units agreed to participate, and in 1994 the registry covered the whole country with 8.8 million inhabitants (today 10 million), the name being changed to Swedvasc [4]. 

The data collected in the registry have been relatively stable over time

The data collected in the registry have been relatively stable over time, but one obvious change has been to include the rapid development within the field of endovascular treatment, where, when the registry started, the only option was percutaneous transluminal angioplasty (PTA).

The aim of this review paper is twofold: to point out the factors which have contributed to the high coverage of the Swedvasc registry and to use registry data to illustrate the development of treatment of abdominal aortic aneurysms (AAA).

## The Swedvasc registry

When the registry started the purposes were as follows:

1. To evaluate results in routine care
2. To create a platform for optimal healthcare planning
3. To create an instrument for evaluation of quality of care
4. To follow the development of new technologies
5. To create a basis for research
6. To create a platform for education

Within the international Vascunet collaboration, founded in 1997 [5] there has been an initiative to perform validation of registries [3, 29]. In 2014 Swedvasc was analyzed and validated by Maarit Venermo, Finland, and Tim Lees, UK, their conclusion being a “highly accurate” registry, where AAA data in the registry had a 96.2% agreement with official hospital statistics [30].

During the life of the registry it would not come as a surprise that the compliance has varied and sometimes been less than optimal with underreporting depending on various factors, such as colleagues in opposition to register, presumed small impact on patient care, lack of time for clinically busy vascular surgeons etc. The underreporting can be on patients and when patients actually are reported, the underreporting may be on various variables, most often preoperative risk factors. So for instance, data on smoking are often missing; however, as the purpose of this communication is to contemplate on a high coverage, factors contributing to this can be summarized as follows:

1. Sweden is a small country with relatively few vascular surgeons (<200) knowing each other with stimulation to cooperate and also inducing some intercollegial pressure to participate.
2. Starting in a small scale and allowing centers to join spontaneously, when they realized that the registry was functioning: voluntarily and not mandatory.
3. A simple data form which had to be a compromise between “covering all” and what was accepted by colleagues to contribute and still detailed enough to be meaningful to be used in analyses.
4. Becoming an organization within the Swedish Society for Vascular Surgery, where all Swedish vascular surgeons are members.
5. Organizing regular meetings for the responsible surgeons with discussions both on the registry and on scientific problems as well as social contacts.
6. Economic support from SKL (Sveriges Kommuner och Landsting, Swedish Association of Local Authorities and Regions). The SKL has ranked Swedvasc to belong to the group of registries in the country with highest quality, among approximately 100 health care registries.
7. Since 2002 Swedvasc is part of Uppsala Clinical Research Centre (UCR) a research organization for registries, particularly involved in cardiovascular diseases and their treatment.
8. The use of Swedvasc for research purposes has been very fruitful and Swedvasc data have until now been used in 15 PhD theses. Research projects can be suggested by all vascular surgeons in Sweden and also other researchers, and the applications are analyzed and approved by a special research committee (approval from regional ethics committees is also required). There are many ways to use Swedvasc data for various research activities:
  - To follow treatment dynamics over time.
  - To assess clinical practice and outcome on regional, national or international level, including benchmarking to identify best practices and assess whether or not clinical practice conforms to current guidelines and evidence [1, 6, 7, 20, 31].
  - To use data for generating hypotheses and for power calculations when designing randomized controlled trials with scientific questions. The registry data has been the basis for several randomized studies, e.g.. Dextroklex, Scamicos, Enoxavasc.
  - To use Swedvasc for registry-based randomization as recently described by the Swedish cardiologist Stefan James, a way to rapidly obtain large patient populations and at a low cost [11]. An ongoing study with this approach is Swedepad, randomizing patients with lower limb ischemia to drug-eluting balloons and stents versus control balloons and bare metal stents.
  - To study rare disorders and rare treatment complications.
  - To perform nested case-control studies.
  - To analyze the relationship between volume and outcome.
  - To perform survival analyses by linking the unique individual patient identification number to the Swedish population registry, where date of death is included within 1 month.
9. To analyze how results from randomized trials can be generalized when diffused into the non-selected general population.
10. Each participating center receives a yearly report of their activity and performances, compared to all other Swedish centers. This is also summarized in a general national report that is publicly available. Moreover, participants have continuous real-time access to the data through the registry homepage.
11. During specialist training colleagues can use their registered data to compare with the overall results in the registry and thus get a feedback and also use the data when applying for a specialist certificate. The feedback has a great educational incentive, which can probably be used even more.
12. Transparency of data, open for patients, which can be used for education and information and stimulate patients to ask the relevant questions on the treatment.

## Treatment of AAA as reflected in Swedvasc

The use of Swedvasc data can be exemplified by analyzing treatment of AAA in the whole of Sweden (from 1994 onwards). It must be emphasized that the information is based only on those patients receiving invasive treatment (open or endovascular surgery). The registry has no data on those dying from ruptures without undergoing treatment (most dying outside hospital) and no data on those with a known AAA, where invasive treatment has not been considered to be indicated.

An AAA repair was performed in >20,000 patients in the period 1994–2014 [17]. There was an increase in surgery for intact AAA, although there were indications of a stoppage in AAA repair in recent years [17]. There was a simultaneous decrease in repair for ruptured cases.

The increased use of endovascular repair (EVAR) was obvious for both manifestations of AAA

The increased use of endovascular repair (EVAR) was obvious for both manifestations of AAA (Table 1). During the period there has been an increase in the age of the patients treated for intact AAA from 71.2 years in 1994–1999 to 72.5 years in 2010–2014 (*p* < 0.001).

**Table 1:** Surgical treatment for abdominal aortic aneurysms (AAA) in Sweden 1994–2016

	1994–1999	2000–2005	2006–2011	2012–2016
*Intact AAA repair*				
EVAR, %	6.1	19.5	47.0	62.7
Rate per 100,000	8.0	9.1	11.3	10.2
*Ruptured AAA repair*				
EVAR, %	0.8	4.9	18.6	37.3
Rate per 100,000	3.7	3.9	3.4	2.4

*EVAR* endovascular aortic repair

The 30-day mortality is shown in Table 2 and this has been significantly influenced to the better over the years (Swedvasc report).

**Table 2:** The 30-day mortality (%) after AAA repair in Sweden 1994–2016

	1994–1999	2000–2005	2006–2011	2012–2016
*Intact AAA repair*				
Open repair	6.2	7.7	3.1	2.5
EVAR	3.4	3.9	1.2	1.1
*Ruptured AAA repair*				
Open repair	45.6	47.3	30.4	28.1
EVAR	68.8	31.1	20.6	21.2

*EVAR* endovascular aortic repair

**Fig. 1:**
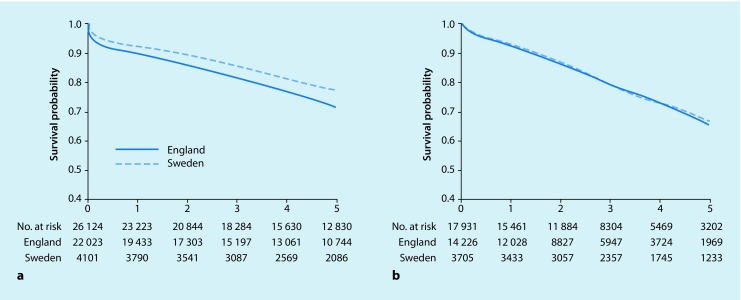
Kaplan-Meier comparison of 5‑year survival after elective abdominal aortic aneurysm repair in Sweden and England with **a** open surgical technique and **b** endovascular repair (adjusted for age and sex). Reproduced from Karthikesalingam et al. [13]

The 5-year survival was 73% after intact AAA repair and 46% after repair of ruptures. Interestingly, the 5‑year outcome was superior in Sweden compared to England for both intact and ruptured AAA (Fig. 1; [12, 13]). Octogenarians and nonagenarians are increasingly being surgically treated, even after ruptures. The old patients with ruptures have a high 90-day mortality (in the order of 50%), but those surviving have a long-term survival only slightly inferior to that of an age-matched general population [23].

The understanding of abdominal compartment syndrome (ACS) has increased over time and thereby how it influences the outcome. In 2008 ACS and decompression laparotomy were introduced as variables in Swedvasc. Thereafter 6612 operations have been analyzed, 20.3% for rupture and in total 52% operated on with EVAR [10]. After repair of ruptures 6.8% developed ACS after open surgery and 6.9% after EVAR. An additional 10.7% were treated with abdomen left open prophylactically after open repair of the aneurysm. After repair of intact AAAs the corresponding figures were 1.6% versus 0.5% (*p* < 0.001). Decompression laparotomy was performed in 77.3% after open repair for ruptured AAAs and in 84.6% after EVAR (NS). In patients treated for ruptured AAA with ACS the 30-day mortality was 42.4% versus 23.5% in those without ACS (*p* < 0.001) and after 1 year the corresponding figures were 50.7% versus 31.8% (*p* < 0.001). The fewer cases with ACS after repair of intact AAA had a significantly higher mortality both at 30 days and at 1 year compared with those without ACS (11.5% vs. 1.8% and 27.5% vs. 6.3%, respectively [both *p* < 0.001]). When ACS developed, renal failure, multiorgan failure and intestinal ischemia were more frequent than in those without ACS (*p* < 0.001). These complications resulted in a significantly longer stay in intensive care units.

During the Swedvasc period, screening for AAA has been successfully introduced since 2006 and from 2015, 65-year-old males in the whole country are invited to attend an ultrasonographic investigation [28]. In this group of patients open repair is significantly more frequent than EVAR as compared to those with AAAs not detected by screening[18]. In patients treated with open repair there was no difference in 30-day, 90-day or 1‑year mortality in AAA detected by screening compared to AAA not detected by screening in controls (1.0% vs. 3.2%, 2.1 vs. 4.5% and 4.1 vs. 5.8%, respectively, all not significant). None of the patients treated with EVAR in either group died within 30 days.

## Discussion

Swedvasc is the first population-based vascular registry with 1‑year follow-up data and with linkage to the unlimited survival data of the population registry and with a very good compliance. The high validity has been verified by external analysis [30]. As most treatment for AAA is performed in patients with concomitant arteriosclerotic disease, a follow-up of at least 1 year seems highly recommendable.

The registry was started by and is still maintained by the vascular surgical profession

Except for the reasons already discussed, one important factor for the almost 100% coverage is probably that the registry was started by and is still maintained by the vascular surgical profession and not being forced from above by authorities, who sometimes are far from the real clinical life. The dedication and idealistic work of all responsible colleagues is a great advantage, which is hereby acknowledged. The high compliance is of utmost importance for reliable data, as patients not reported to the registry tend to have a worse outcome [9]. To keep validity high, both internal and external regular validation should be undertaken with a continuous feedback to the participating centers and surgeons [15, 27].

Sometimes there is a vivid discussion between proponents for registries as opposed to randomized trials. It is our firm view that both types are needed. When interpreting data it is important to be aware that both strategies have their strengths and weaknesses, and the two research strategies are in fact complementary. The generalizability of results from randomized trials must always be surveyed when being used in the whole population at risk, and that is made in validated registries, reflecting the real-world situation.

One valuable step in recent years has been the cooperation with registries in other countries in the Vascunet network with the possibility of direct comparisons and where observations of differences can stimulate improvements in care and to design scientific projects [8, 19]. At present the network includes 12 registries from Europe, New Zealand and Australia.

Several changes have been observed over time within the registry both in treatment and outcome, and we have chosen AAA to exemplify the temporal development from 1994, a period when Swedvasc has covered the whole Swedish population. A finding, which is important to emphasize, is the decrease in postoperative mortality, another is the increasing use of EVAR. Similar trends have been reported in a German registry [25, 26] as well as in the Vascunet collaboration [6].

If and how the use of a registry influences the outcome is a question not easily answered. The situation is complex with several potential components, such as changes in patient selection, technical developments, better anesthesia and intensive care, more optimized risk factor management, especially the introduction of pharmacological and other treatment and there may even be factors that we presently are not aware of. Observations within the registry may lead also to changes, one example being the AAA treatment in the UK. In an early Vascunet comparison the postoperative mortality after elective AAA repair was significantly higher in the UK than in the other participating countries [22]. This lead to an in-depth analysis of the situation, the development of a Vascular Services Quality Improvement Program (VSQIP) and stimulated the formation of comprehensive vascular networks. One conclusion was too many operations in small units, and after correcting this situation by centralization the UK mortality does not differ from that in the other Vascunet countries [21]. Another observation within Swedvasc is the increasing number of treated patients, and this is seen in spite of the decreasing AAA prevalence in Sweden [16, 24]. The explanation can be at least two-fold, the introduction of screening, diagnosing more small and asymptomatic aneurysms, and the introduction of EVAR with more liberal indications, for instance treating more older and more patients with comorbidities, where open surgery was not an option before the endovascular era.

An important change over the years is the decrease in treatment for ruptures with the much higher risks than when treating electively. This can be explained by a combination of a falling prevalence of the disease, the introduction of screening, and on the increasing number of elective operations. If the development of AAA treatment will influence the total mortality in the disease is unknown and will be difficult to investigate with the extremely low autopsy rate we have today (<10%). Most patients with ruptures die outside hospital or in non-surgical wards [2, 14], and moreover the registry has no information on the turndown rate for surgery.

## Conclusion

Many factors have contributed to the high coverage of the Swedvasc registry, and that has made it possible to study the development of treatment and outcome including complications, which in this article is exemplified with abdominal aortic aneurysms.
